# A community resource for high-throughput quantitative RT-PCR analysis of transcription factor gene expression in *Medicago truncatula*

**DOI:** 10.1186/1746-4811-4-18

**Published:** 2008-07-08

**Authors:** Klementina Kakar, Maren Wandrey, Tomasz Czechowski, Tanja Gaertner, Wolf-Rüdiger Scheible, Mark Stitt, Ivone Torres-Jerez, Yongli Xiao, Julia C Redman, Hank C Wu, Foo Cheung, Christopher D Town, Michael K Udvardi

**Affiliations:** 1 Max-Planck Institute of Molecular Plant Physiology, Am Mühlenberg 1, 14476 Potsdam-Golm, Germany; 2 The J. Craig Venter Institute, 9704 Medical Center Drive, Rockville, MD, 20850, USA; 3 The Samuel Roberts Noble Foundation, 2510 Sam Noble Parkway, Ardmore, OK, 73401, USA

## Abstract

**Background:**

*Medicago truncatula *is a model legume species that is currently the focus of an international genome sequencing effort. Although several different oligonucleotide and cDNA arrays have been produced for genome-wide transcript analysis of this species, intrinsic limitations in the sensitivity of hybridization-based technologies mean that transcripts of genes expressed at low-levels cannot be measured accurately with these tools. Amongst such genes are many encoding transcription factors (TFs), which are arguably the most important class of regulatory proteins. Quantitative reverse transcription-polymerase chain reaction (qRT-PCR) is the most sensitive method currently available for transcript quantification, and one that can be scaled up to analyze transcripts of thousands of genes in parallel. Thus, qRT-PCR is an ideal method to tackle the problem of TF transcript quantification in Medicago and other plants.

**Results:**

We established a bioinformatics pipeline to identify putative TF genes in *Medicago truncatula *and to design gene-specific oligonucleotide primers for qRT-PCR analysis of TF transcripts. We validated the efficacy and gene-specificity of over 1000 TF primer pairs and utilized these to identify sets of organ-enhanced TF genes that may play important roles in organ development or differentiation in this species. This community resource will be developed further as more genome sequence becomes available, with the ultimate goal of producing validated, gene-specific primers for all Medicago TF genes.

**Conclusion:**

High-throughput qRT-PCR using a 384-well plate format enables rapid, flexible, and sensitive quantification of all predicted Medicago transcription factor mRNAs. This resource has been utilized recently by several groups in Europe, Australia, and the USA, and we expect that it will become the 'gold-standard' for TF transcript profiling in *Medicago truncatula*.

## Background

Legumes are second only to grasses in agricultural importance [1]. They are a mainstay of sustainable agricultural systems because of their ability to reduce atmospheric nitrogen (N_2_) to ammonia via a symbiosis with bacteria called rhizobia. This provides legumes and subsequent crops with a free and renewable source of nitrogen in lieu of expensive, environmentally-unfriendly fertilizers. Development and differentiation of root nodules, the organ that accommodates nitrogen-fixing rhizobia in legumes, is orchestrated by transcription factors [2-9]. Transcription factors are DNA-binding proteins that regulate the transcription of most, if not all genes [10]. As a result, TFs play central roles in all aspects of plant biology, including development and differentiation of organs and adaptive responses to changes in the environment [11]. Transcription factors as a whole are an important target of plant research because they are a key to understanding the regulation of important plant processes as well as potential tools to optimize these processes for agriculture.

The importance of TFs in plant biology is reflected by the fact that approximately 5% of all plant genes encode such proteins [10]. Thus, even species with relatively small genomes, such as *Arabidopsis thaliana *contain thousands of TF genes [10]. This presents a real challenge for systematic approaches to decipher the function of TF genes in plants. Classical, 'forward' genetics has uncovered the roles of perhaps a hundred TF genes in Arabidopsis [12] and far fewer in other species [11]. Reverse-genetic approaches, using T-DNA insertion mutants for instance [13], provide a means to decipher in a systematic and relatively rapid manner the function of TF genes/proteins, although gene-redundancy often stymies this enterprise [12]. Another stumbling-block is that phenotypes associated with non-redundant TFs may be subtle in nature.

Transcript profiling can help to uncover the functions of TF genes/proteins by revealing where and when in a plant TF genes are expressed. This information can help direct our attention to particular organs, developmental stages, or conditions under which aberrant phenotypes might become apparent in a TF mutant of interest.

*Medicago truncatula *is a model legume species that is currently the focus of an international genome sequencing effort [14]. Several generations of cDNA [15] and oligonucleotide arrays [16] have been developed for transcriptome analysis of *Medicago truncatula*, including most-recently an Affymetrix GeneChip that contains 51,000 probe-sets representing a large proportion of all the genes in this species [17]. While these tools now provide a means to measure the transcriptional output of a large proportion of genes in Medicago, inherent limitations in the sensitivity of hybridization-based technologies [18] mean that transcripts of a substantial number of genes cannot be detected even when probes for these transcripts are present on the array/chip. Furthermore, expansion of arrays to encompass novel genes uncovered by genome sequencing is not a trivial task. An alternative to arrays that is 2–3 orders of magnitude more sensitive and more flexible in terms of expansion to encompass novel genes is quantitative reverse transcription-polymerase chain reaction (qRT-PCR). Platforms for qRT-PCR analysis of thousands of Arabidopsis and rice TF genes have been developed by us and others [19,20], and utilized to identify TF genes involved in Arabidopsis responses to nutrient stress and pathogen attack [21-24]. Here we describe a bioinformatics pipeline to identify putative TF genes in *Medicago truncatula *and to design gene-specific oligonucleotide primers for qRT-PCR analysis of all predicted TF transcripts. Over 1000 TF primer pairs were tested and used to identify sets of organ-enhanced TF genes that may play important roles in organ development or differentiation in this species.

## Results and Discussion

### Identification of putative transcription factors

TF protein families are generally defined by the type(s) of DNA-binding domain they contain and putative TF genes are often identified on the basis of DNA sequences that encode known DNA-binding domains [10,11,25,26]. We utilized this approach to identify putative TFs of Medicago amongst the set of proteins predicted from genomic sequence by the International Medicago Genome Annotation Group (IMGAG). Proteins of IMGAG release 1, which contained over 40,000 predicted proteins, were screened for the presence of known or presumed DNA-binding domains (Table 1), using INTERPRO [27]. Medicago proteins containing putative DNA binding domains and other domains associated with TFs were then used as query sequences in WU-BLASTX [28] which included searches of both the non-redundant DNA database of NCBI [29] and the well-curated protein database, UniProt [30] to check annotations of related proteins in support of tentative Medicago TF assignments. This process resulted in a list of 1045 putative TF genes (see Additional file 1). We utilized genomic sequences rather than the large collection of partial cDNA sequences present in Expressed Sequence Tag (EST) databases for Medicago as the starting point for TF gene discovery because protein sequences derived from genomic sequence are more complete and the set of IMGAG proteins essentially contains no redundancy. Although identification of the 'complete' set of Medicago TFs from IMGAG-annotated proteins will only be possible upon completion of genome sequencing, we expect little or no redundancy in the protein set targeted by our primer set. This approach avoids wasting money on redundant primer sets and re-organization of primers when redundancy is detected, both of which would have been inevitable if we chose to use ESTs in addition to genomic sequences to identify Medicago TFs.

**Table 1:** Classification of putative transcription factors of Medicago into families and sub-families

**TF Family**	**No. of ** **Genes**	**Characteristic Domain ** **(InterPro No.)**	**Domain** **Function**	**Domain Description**
MYB/HD-like	76	IPR001005, IPR009057	D	Myb, DNA-binding; homeodomain like
MYB	58	IPR001005	D	Myb, DNA-binding
C_2_H_2 _(Zn)	64	IPR007087	NA	Zn-finger, C2H2 type
**AP2/EREBP**	**55**	**IPR001471**	**D**	**Pathogenesis-related transcriptional factor and ethylene response factor**
BHLH	49	IPR001092	D	Basic helix-loop-helix dimerisation region bHLH
HD-like	50	IPR009057	D	Homeodomain like
HD family		IPR001356	D	Homeobox
HD	25			
HD-ZIP	5	IPR006712	P	HD-ZIP protein, N terminus
HD-PHD finger	2	IPR001965	P	Zn-finger like, PHD-finger
MADS	48	IPR002100	D	TF, MADS-box
BZIP	41	IPR004827	D	Basic Leu zipper (bZIP) TF
PHD	34	IPR001965	P	Zn-finger like, PHD-finger
**WRKY family**		**IPR003657**	**D**	**DNA-binding WRKY**
**WRKY**	**29**			
**LLR WRKY**	**1**	**IPR001611**		**Leu-rich repeat**
**ABI3/VP1**	**29**	**IPR003340**	**D**	**TF B3**
**NAC**	**29**	**IPR003441**	**D**	**No apical meristem (NAM) protein**
C_3_H-type1(Zn)	27	IPR000571	D	Zn-finger, C-x8-C-x5-C-x3-H type
**ARF**	**23**	**IPR003340, IPR010525, IPR011525**	**D**	
JUMONJI	20	IPR003347	D	TF jumonji, jmjC
**GRAS**	**19**	**IPR005202**	**P**	**GRAS TF**
HMG	15	IPR000637	D	HMG-I and HMG-Y, DNA binding
AS2	14	IPR004883	P	Lateral organ boundaries
C_2_C_2 _(Zn)				
**Dof**	**13**	**IPR003851**	**D**	**Zn-finger, Dof type**
GATA	7	IPR000679	D	Zn-finger, GATA type
**CO-like**	**6**	**IPR000315**	**D**	**Zn-finger, B-box**
**YABBY**	**5**	**IPR006780**	**D**	**YABBY protein**
CCAAT-HAP3 type	12	IPR003958	D	TF CBF/NF-Y/archaeal histone
**GRF**	**8**	**IPR010666**	**D**	**Zn-finger, GRF type**
**SBP**	**8**	**IPR004333**	**D**	**SBP**
**EIL**	**7**	**IPR006957**	**D**	**Ethylene insensitive 3**
LIM	7	IPR001781	P	Zn-binding protein, LIM
SNF2	6	IPR000330	D	SNF2 family N-terminal domain
E2F/DP	5	IPR003316	D	TF E2F/dimerisation partner (TDP)
**TCP**	**5**	**IPR005333**	**D**	**TCP TF**
**FHA**	**5**	**IPR000253**	**D**	**Forkhead-associated**
ARID	4	IPR001606	D	AT-rich interaction region
HSF	4	IPR000232	D	Heat shock factor (HSF)-type, DNA binding
**AUX/IAA**	**3**	**IPR003311**	**D**	**AUX/IAA protein**
**SRS**	**3**	**IPR006510**	**D**	**Zn-finger, LRP1 type**
TUB	3	IPR000007	D	Tubby
ZIM	3	IPR010399	D	ZIM
DDT	3	IPR004022	D	DDT
**ZF-HD**	**2**	**IPR006455**	**D**	**Homeobox domain, ZF-HD class**
**MBF1**	**2**	**IPR001387**	**D**	**Helix-turn-helix type 3**
**S1Fa-like**	**2**	**IPR006779**	**D**	**DNA binding protein S1FA**
**CAMTA**	**2**	**IPR005559**	**D**	**CG-1**
**LFY**	**1**	**IPR002910**	**D**	**Floricaula/leafy protein**
**Nin-like**	**1**	**IPR003035**	**D**	**Plant regulator RWP-RK**
**TAZ**	**1**	**IPR000197**	**P**	**Zn-finger, TAZ-type**

**Potentially novel plant TFs and transcriptional regulators**				

CCHC (Zn)	112	IPR001878	NA	Zn-finger, CCHC-type
RR	16	IPR001789, IPR011006	RD	Response regulator receiver
DHHC (Zn)	14	IPR001594	D or P	Zn-finger, DHHC-type
HTH				
FIS	11	IPR002197	D	Helix-turn-helix, Fis-type
AraC	2	IPR000005	D	Helix-turn-helix, AraC type
BTB/POZ	7	IPR000210	P	BTB
TTF-type (Zn)	6	IPR006580	D	Zn-finger, TTF-type
BD	6	IPR001487	P	Bromodomain
Lambda-DB	3	IPR010982	D	Lambda_DNA_bd
TrpR	3	IPR010921	D	Trp repressor/replication initiator
TPR	3	IPR001440	P	Tetratricopeptide TPR_1
KRAB-box	2	IPR001909	P	KRAB box
NRs	2	IPR008946	LBD	Steroid nuclear receptor, ligand binding
R3H	2	IPR001374	NA	Single-stranded nucleic acid binding R3H
YEATS	2	IPR005033	TA	YEATS
U1-type (Zn)	2	IPR003604	NA	Zn-finger, U1-type
A20-like	2	IPR002653	P	Zn-finger, A20-type
Euk_TF	1	IPR008917	D	Euk_TF, DNA binding
NGN	1	IPR006645	D	NGN
p53-like	1	IPR008967	D	p53-like TF, DNA binding
SSB protein	1	IPR011344	D	Single-strand binding protein
ssDB TR	1	IPR009044	D	Single-strand DNA binding transcriptional regulator
TCoAp15	1	IPR003173	D	Transcriptional coactivator p15
BED-type (Zn)	1	IPR003656	D	Zn-finger, BED-type predicted
TCoA	1	IPR009255	TA	Transcriptional coactivation
Tc/PD	1	IPR001533	TA	Transcriptional coactivator

IMGAG (International Medicago Gene Annotation Group)-proteins were classified as putative TFs if they contained characteristic DNA-binding or other characteristic TF domains and if annotations of matching proteins obtained by BLAST searches were consistent with such a classification. TF families previously identified in plants are presented in the first part of the table while potentially novel plant TF families, which were identified by the presence of domains associated with TFs and other transcriptional regulators outside the plant kingdom, are presented in the latter part of the table. D = DNA binding domain; P = protein-protein interaction domain; NA = nucleic acid (DNA and RNA) binding domain; RD = receiver domain; LBD = ligand binding; transcriptional co-activator. Plant-specific TF families and sub-families are indicated in bold (according to [12])

### PCR primer design

To ensure maximum specificity and efficiency during PCR amplification of TF cDNA under a standard set of reaction conditions, a stringent set of criteria was used for primer design. This included predicted melting temperatures (*T*_m_) of 58°C to 61°C, limited self-complementarity and poly-X, and PCR amplicon lengths of 100–150 base pairs (bp). Secondary hits were minimized by aligning primer candidates to all known Medicago sequences via WU-BLAST [28] and eliminating primer pairs with multiple potential hits.

### PCR primer testing: gene-specificity and amplification efficiency

PCR primers were tested on Medicago cDNA free of genomic DNA contamination as follows. First, total RNA was extracted from various organs using Trizol reagent (Invitrogen GmbH, Karlsruhe, Germany), which yielded high quality RNA as judged by gel electrophoresis and by Agilent 2100 BioAnalyser using RNA 6000 Nano Chips (Agilient Technologies, Waldbronn, Germany). Typical RNA yields ranged from 0.5–1.0 μg RNA/mg fresh mass for nodules and leaves, respectively. Isolated RNA was treated with DNAse I (Ambion, product number 1907) to remove all contaminating genomic DNA, and this was always confirmed by PCR using primers to non-coding regions of the *Ubiquitin *gene (TC102473; AC137828-19.4). After inactivation of DNAse I, RNA was reverse transcribed using SuperScript III reverse transcriptase (Invitrogen GmbH, Karlsruhe, Germany) and oligo-dT_12–18 _to prime the reaction.

**Figure 1:**
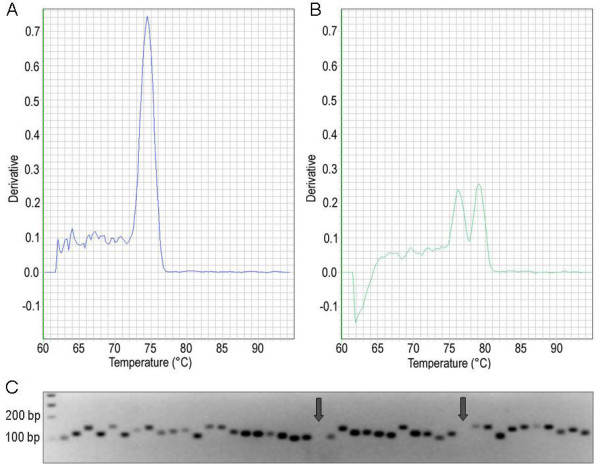
**Specificity of transcription factor PCR primers**. Specificity was confirmed by dissociation curves with a single peak (A) while double peaks (B) indicated off-target ampification. The derivative of fluorescence intensity is shown on the y-axis. Separation of PCR products on 3% (v/w) agarose gels following electrophoresis (C) confirmed the presence of unique amplicons of the expected size for most reactions. Few reactions yielded no products (indicated by arrow).

Specificity of PCR primers was assessed in three ways: by melting curve analysis of PCR reaction products; by separating the products of all reactions via electrophoresis in 3% agarose gels; and by sequencing a sub-set of PCR reaction products (Figure 1). 94.5% (998/1045) of primer pairs gave unique PCR products of the expected size. Only 3.3% (34/1045) of primer pairs yielded no product and 2.2% (23/1045) gave non-specific products. (see Additional file 1). Sequencing was performed on 178 randomly-chosen PCR products amplified from a 1:1 mixture of leaf and root cDNA. In the vast majority of cases (92.7% or 165/178), the sequence of the PCR product was identical to that of the intended target gene. In 5.1% of cases, the amplicon sequence matched multiple related genes, including the target gene, while in only 2.2% of cases the amplicon sequence did not match the target gene sequence.

**Figure 2:**
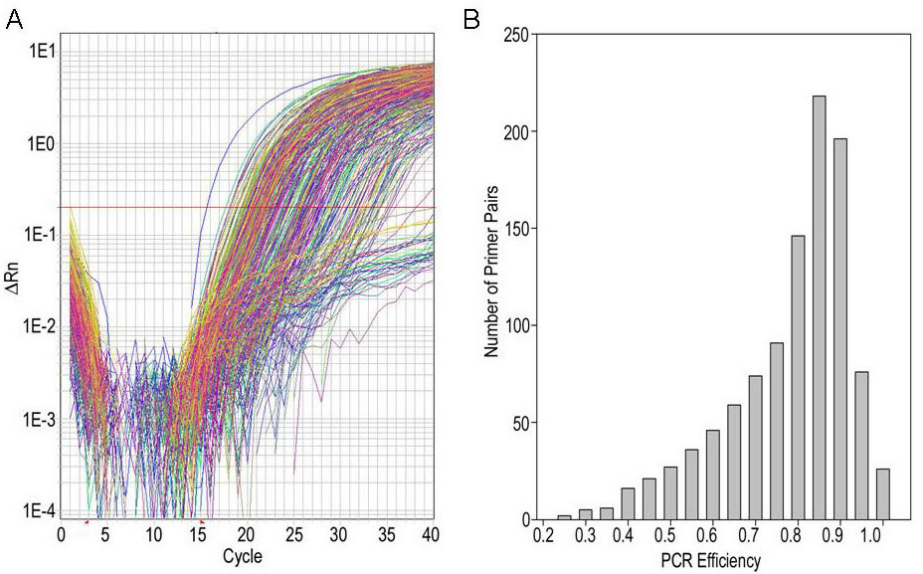
**Amplification efficiency of transcription factor-specific primer pairs**. Typical real-time RT-PCR amplification plots of 384 TF genes (left) and distribution of PCR efficiencies for all 1045 TF primer pairs (right).

Ideally, PCR results in an exact doubling of the amount of dsDNA after each temperature cycle. In practice, however, this is generally not the case because the reactions are less than 100% efficient. Primer sequences can affect PCR efficiency, so we determined the efficiency of each TF primer pair from amplification plots, using LinRegPCR software [31]. First, the correlation coefficient derived from linear regression analysis of each amplification plot (e.g. see Figure 2) was used to assess the 'quality' of each reaction, and all reactions with an R^2 ^< 0.990 were excluded from further analysis (10.6% of reactions). Next, average PCR efficiencies (E) were computed for each individual primer pair across all analyzed samples. 53.4% (558 TF genes) displayed PCR efficiencies greater than 0.80, while 39.7% (415 TF genes) had efficiencies between 0.51–0.80. Only 2.6% (27 TF genes) had mean E values below 0.4; these all yielded R^2 ^< 0.99 in LinRegPCR analysis and mostly represented reactions that lacked detectable fragment amplification (C_T _> 40) or that generated unspecific PCR products (Figure 2; see Additional file 1). A similar range of PCR efficiencies were determined for Arabidopsis and rice TF primers previously [19,20].

### Selection of reference genes

**Figure 3:**
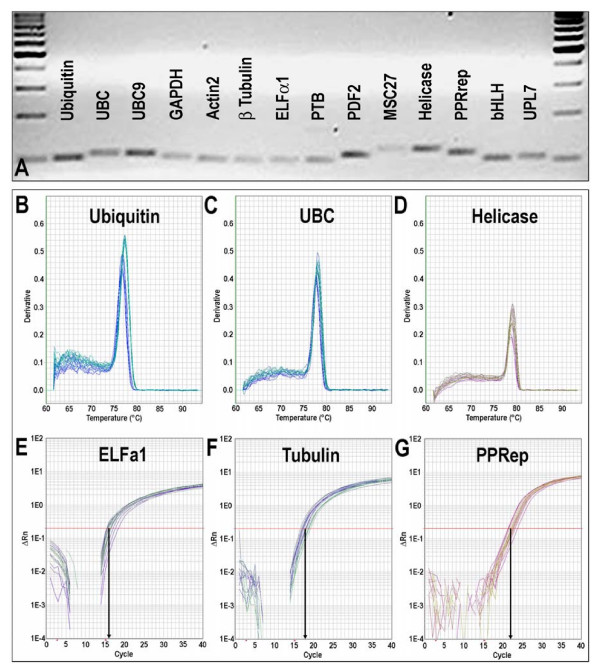
**Specificity, efficiency, and reproducibility of PCR primers designed to amplify reference gene transcripts**. Specificity of primers was confirmed by the presence of unique amplicons of the expected size following electrophoresis on 3% (v/w) agarose gels (A) and by dissociation curves with a single peak (B to D). Typical real-time RT-PCR amplification plots of three reference gene transcripts (E to G).

**Figure 4:**
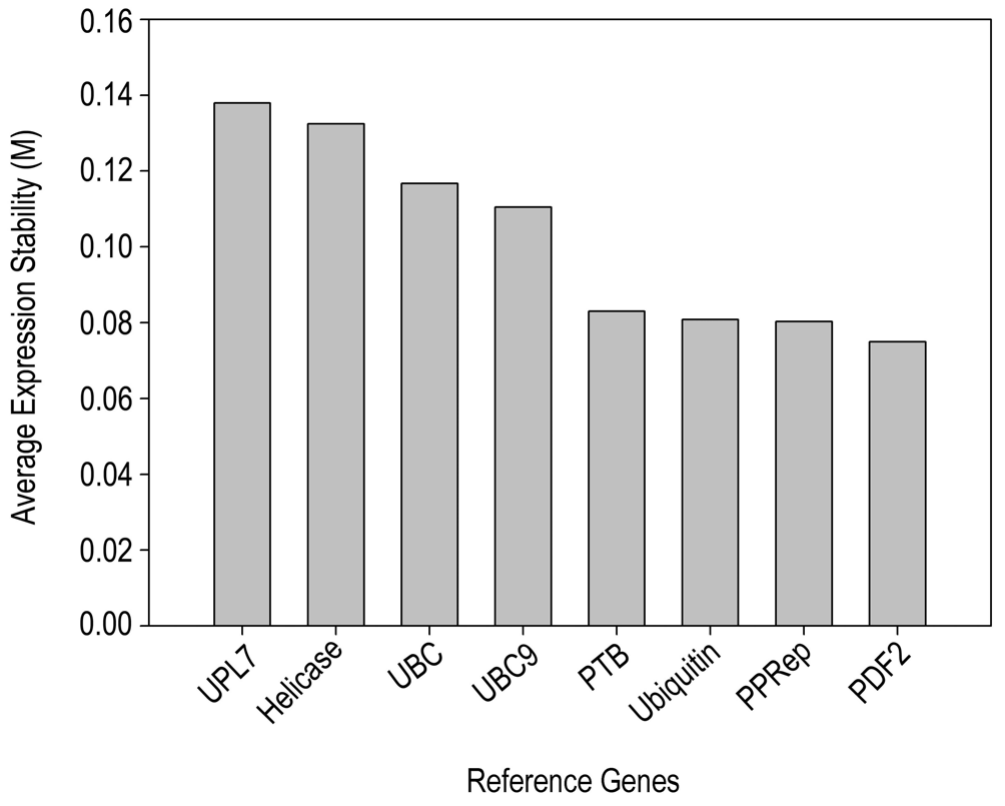
**Ranking of 8 reference genes in *M. truncatula***. Transcript levels of all 8 genes were measured by qRT-PCR, using 18 independent cDNA preparations from six different organs with three replicate measurements of each cDNA preparation. A low value for the average expression stability M, as calculated by geNORM software, indicates more stable expression throughout the various organs.

Reference genes with stable expression/transcript levels throughout development and in the face of environmental challenge are crucial for the normalization of expression data of other genes. Potentially useful reference genes were chosen based on published data for Medicago (e.g. *Msc27 *[32]) and *Arabidopsis thaliana *[33]. The closest Medicago homologues of Arabidopsis genes were identified by BlastN [29]. Gene-specific primer pairs for the genes encoding elongation factor 1α (EST317575), glyceralaldehyde-3-phosphate dehydrogenase (MtC00030_GC; CT573421_3.4), β-tubulin (TC106341), Pentatricopeptide repeat protein (TC96273), actin2 (TC107326; AC137836_27.5), Ubiquitin (TC102473; AC137828-19.4), Helicase (CB892427), and the genes *PDF2 *(TC107161), *UPL7 *(TC111218), *PTB *(TC111751), *UBC *(AW686873), *bHLH *(CX538576), and *UBC9 *(TC106312) were designed using the criteria described above (Table 2). The specificity of PCR primers was tested using 18 first-strand cDNAs from six different organs of *Medicago *(three biological replicates each). All primer pairs produced a single PCR product of the expected size, as shown by gel electrophoresis and unique dissociation curves generated by the PCR machine after 40 cycles (Figure 3). To determine which reference genes were best suited for transcript normalisation, we used the software geNORM [34], which uses pair-wise comparison and geometric averaging across a matrix of biological samples to determine gene expression stability (M; [35]). The genes *PDF2*, *PPRep*, *Ubiquitin, and PTB h*ad the lowest M (greatest transcript stability) and, therefore, were judged to be the best reference genes for this diverse set of developmental samples (Figure 4; Table 2).

**Table 2:** Medicago reference genes and primers for qRT-PCR

**Gene Name**	**TC**	**Accession** **Number**	**Forward/Reverse Primer (5'-3')**	**PCR ** **Product** ** Size (bp)**	**PCR ** **efficiency** ** (E)**	**R**^2^
β Tubulin	TC106341	N	TTTGCTCCTCTTACATCCCGTG / GCAGCACACATCATGTTTTTGG	100	1.08	1.00
PPRrep	TC96273	N	GGAAAACTGGAGGATGCACGTA / ACAAGCCCTCGACACAAAACC	100	0.93	1.00
PDF 2	TC107161	N	GTGTTTTGCTTCCGCCGTT / CCAAATCTTGCTCCCTCATCTG	100	0.99	1.00
bHLH	CX538576	N	TAGCGAGTACCATGATGCCAGA / GCGCCTCTTTTGTTTTCAGC	100	0.89	1.00
UBC	AW686873	N	CTGACAGCCCACTGAATTGTGA / TTTTGGCATTGCTGCAAGC	100	0.96	1.00
PTB	TC111751	N	CGCCTTGTCAGCATTGATGTC / TGAACCAGTGCCTGGAATCCT	100	0.85	1.00
Ubiquitin	TC102473	AC137828_19.4	GCAGATAGACACGCTGGGA / AACTCTTGGGCAGGCAATAA	100	0.95	1.00
UBC9	TC106312	AC137602_2.4	GGTTGATTGCTCTTCTCTCCCC / AAGTGATTGCTCGTCCAACCC	100	1.13	0.99
Helicase	CB892427	N	GTACGAGGTCGGTGCTCTTGAA / GCAACCGAAAATTGCACCATAC	100	0.91	1.00
ELF1α	EST317575	N	GACAAGCGTGTGATCGAGAGATT / TTTCACGCTCAGCCTTAAGCT	100	0.68	0.98
UPL7	TC111218	N	CCAGTTGTTCTCGTGGTCCATT / CCTCCAATTGTCGCCCAAA	100	0.93	1.00
GAPDH	MtC00030_GC	CT573421_3.4	TGCCTACCGTCGATGTTTCAGT / TTGCCCTCTGATTCCTCCTTG	100	1.04	0.99
Actin2	TC107326	AC137836_27.5	TCAATGTGCCTGCCATGTATGT / ACTCACACCGTCACCAGAATCC	100	1.12	0.99
MSC27	X63872 (*M. sativa*)	N	GTTGAAGTAGACATTGGTGCTAACG / AGCTGAGTCATCAACACCCTCAT	100	0.76	0.99

Mean PCR efficiency (E) was determined from three biological replicates of each of six organs, using LinRegPCR [31], which also yielded mean R^2^. N, no corresponding GenBank accession number.

### Identification of organ-enhanced TFs of Medicago

To get an overview of TF gene expression in *Medicago truncatula *and to identify TFs induced in specific organs, we used the real-time RT-PCR platform described above. Transcript profiling was performed on six different organs of *Medicago *(leaves, stems, flowers, pods, roots, and nodules) with three independent biological replicates for each (see Additional file 2). The fraction of genes for which transcripts were detected within 40 cycles ranged from 77.2% in leaves to 90.8% in pods. Transcripts from nearly all putative TF genes (96.8% or 1011/1045) were detected in at least one organ. Genes were called detected if they were expressed in at least two biological replicates with a C_T _< 40. Approximately half of all TF genes exhibited differential expression during plant development, based on significant differences (p ≤ 0.05) in transcript levels between organs. Few TF genes (1.19% or 12/1011) were expressed exclusively in vegetative organs (leaves, stems, roots or nodules), and even fewer (0.5% or 5/1011) were expressed only in reproductive organs (flowers or pods). A relatively small number of TF genes exhibited greater than ten-fold ratios in expression level in one organ compared to any other organ (Table 3). For comparison, we have included gene expression ratios derived from Affymetrix array data from the same RNA samples. While there is reasonable qualitative agreement between gene expression ratios obtained using the two methods, the lack of quantitative agreement is likely due to the limited sensitivity and low signal to noise ratio near the detection limit of Affymetrix arrays [19]. The genes listed in Table 3 may control development and/or differentiation in Medicago and are interesting targets for future research.

**Table 3:** Organ-enhanced TF genes

**A **Root-enhanced TF genes identified by real-time RT-PCR											
	**Real-time RT-PCR Expression Ratio**					**Affymetrix Expression Ratio**					

**Accession Number**	**R/L**	**R/S**	**R/F**	**R/P**	**R/N**	**R/L**	**R/S**	**R/F**	**R/P**	**R/N**	**Affymetrix Chip ID**

AC140721_12.1	121.5	320.1	1623.4	2676.5	**64.4**	161.2^b^	144.5^b^	219.2^b^	158.1^b^	99.9	Mtr.50075.1.S1_s_at
AC135101_25.1	312.2	438.0	291.6	787.0	**61.3**	n	n	n	n	n	n
AC140721_14.1	1204.9	751.2	956.6	6296.6	**32.6**	n	n	n	n	n	n
AC140031_3.1	275.4	1157.3	923.4	258916.9^a^	**31.1**	0.4	0.7	0.8	0.9	2.2	Mtr.47227.1.S1_s_at
AC140721_13.1	39.9^a^	179.8	614.8	935.1^a^	**26.5**	35.9	48.1^b^	41.2	44.2^b^	39.5	Mtr.50074.1.S1_at
AC140031_7.1	360.2^a^	830.6	1542^a^	6868.8	**19.7**	0.7^b^	1^b^	1.1^b^	1^b^	0.9^b^	Mtr.47229.1.S1_at
AC146574_6.1	1095.7	**11.9**^a^	48.8	51.8	88.8	1.2^b^	1.1^b^	1.3^b^	1.1^b^	1.2^b^	Mtr.40781.1.S1_s_at
AC125478_13.7	1117.4	7008.0	3264.9^a^	7256.6	**11.6**	211.5^b^	248.8^b^	296.4^b^	228.7^b^	45.3	Mtr.15416.1.S1_at
AC125478_7.2	1052.3	503.3	938.4^a^	2754.5	**11.2**	n	n	n	n	n	n
AC122726_21.111	50.0	48.0	20943.9	2658.6	**10.4**	74.3^b^	76.4^b^	72.8^b^	82.8^b^	26.6	Mtr.15568.1.S1_s_at

**B **Nodule-enhanced TF genes identified by real-time RT-PCR											

	**Real-time RT-PCR Expression Ratio**					**Affymetrix Expression Ratio**					

**Accession Number**	**N/L**	**N/S**	**N/F**	**N/P**	**N/R**	**N/L**	**N/S**	**N/F**	**N/P**	**N/R**	**Affymetrix Chip ID**

AC148816_3.2	9966.7	1826.2	6262.9	21709.1	**662.7**	434.6^b^	516.8^b^	455^b^	521.7^b^	551.4^b^	Mtr.14503.1.S1_at
AC147774_3.2	**55.2**^a^	466.7	726.3	881.2	498.5	17.7^b^	16^b^	17.8^b^	19.1^b^	16.1^b^	Mtr.19554.1.S1_at
AC138056_33.241	43.9	93.2	18.3	17.3	**13.1**	0.9^b^	0.8^b^	0.8^b^	1.1^b^	1.1^b^	Mtr.17993.1.S1_at
AC124214_39.2	121.8	92.1	44.8	64.9	**10.5**	n	n	n	n	n	n

**C **Pod-enhanced TF genes identified by real-time RT-PCR											

	**Real-time RT-PCR Expression Ratio**					**Affymetrix Expression Ratio**					

**Accession Number**	**P/L**	**P/S**	**P/F**	**P/N**	**P/R**	**P/L**	**P/S**	**P/F**	**P/N**	**P/R**	**Affymetrix Chip ID**

AC143340_4.7	154.1	**44.8**	173.3	180.3^a^	407.3	1.6^b^	1.8^b^	1.7^b^	1.7^b^	1.7^b^	Mtr.17931.1.S1_at

**D **Flower-enhanced TF genes identified by real-time RT-PCR											

	**Real-time RT-PCR Expression Ratio**					**Affymetrix Expression Ratio**					

**Accession Number**	**F/L**	**F/S**	**F/P**	**F/N**	**F/R**	**F/L**	**F/S**	**F/P**	**F/N**	**F/R**	**Affymetrix Chip ID**

AC129092_13.1	**40.3**	383.3	65.1	1683.9	318.1	24.2	47.1^b^	38.2	45.4^b^	55.1^b^	Mtr.16432.1.S1_at
AC148485_10.1	476.2	203.7	**27.2**	174.6	44.5	36.5^b^	32.9^b^	22.0	34.7^b^	29.7^b^	Mtr.20392.1.S1_at
AC140915_20.1	169.5	101.1	**18.6**	481.0	178.7	1.4^b^	1.4^b^	1.3^b^	1.3^b^	1.6^b^	Mtr.51688.1.S1_at
AC141107_50.2	38.2	33.0	**17.9**	114.0	97.4	n	n	n	n	n	n
AC144731_15.21	64.4	44.3	33.6	61.5	**16.3**	0.6^b^	0.6^b^	0.8^b^	0.8^b^	0.8^b^	Mtr.19093.1.S1_at
AC150978_12.1	235.3	198.5	48.4	**14.0**	16.9	n	n	n	n	n	n
AC141107.5.61	261.1	127.8	**13.3**	96.4	71.5	0.9^b^	0.9^b^	0.8^b^	0.1^b^	1.1^b^	Mtr.51651.1.S1_at
AC157472_19.1	60.2^a^	92.1	**13.2**	61.5^a^	113.6	n	n	n	n	n	n
AC148527_19.141	188.4	42.1	17.3	26.5	**13.0**	n	n	n	n	n	n
AC144726_6.1	254.7	55.2	**10.9**	112.7^a^	754.7	59.2	73.7^b^	7.6	89.2	97.9^b^	Mtr.19024.1.S1_at
AC157488_16.1	7053.2^a^	141.1	18.7	81.8	**10.7**	n	n	n	n	n	n
AC123899_15.181	**10.2**	92.2	19.1	62.8	24.9	0.9^b^	0.5^b^	0.8^b^	0.1^b^	0.8^b^	Mtr.52015.1.S1_at

A TF gene was considered organ-enhanced if transcript levels for that gene were more than 10-fold higher in one organ than in any other organ. Transcript ratios were calculated using the mean of three biological replicates for each organ. Data from qRT-PCR are compared with data for the same RNA samples obtained from Affymetrix Gene chips [38]. Affymetrix data were normalized using the Robust Multiarray Average (RMA) method, as described by [38], prior to calculation of ratios. Data in bold represent the lowest transcript ratio of the corresponding gene across all organs. n = not present on Affymetrix chip; a = Ct > 40 in two or three biological replicates of denominator organ; b = transcript called 'absent' by Affymetrix software in two or three biological replicates of denominator organ. L = leaf; S = stem; P = pod; F = flower; R = root; N = nodule.

## Conclusion

We have established a flexible platform for high-throughput qRT-PCR analysis of Medicago TF gene expression that is based on gene-specific primers arrayed in 384-well plates and SYBR^® ^Green detection of gene-specific PCR amplicons. Currently, the platform has primer pairs for 1045 TF genes and we have plans to extend this to all predicted Medicago TF genes as genome sequencing progresses. At this stage, the resource has been utilized by several groups in Europe, Australia, and the USA, and we expect it will become the 'gold-standard' for TF transcript profiling in *Medicago truncatula*.

## Methods

### Plant material and growth conditions

*Medicago truncatula *cv. Jemalong, line A17 wild type plants were vernalized for 3 days in the dark at 4°C on sterile, wet filter paper. Germinated seedlings were transferred to pots containing Turface (BWI Texarcana, Texarcana, TX). Plants were grown in growth chambers under a 16 h day and 8 h night regime, at 200 μE light intensity, 24°C and 40% relative humidity.

Vegetative organs (leaves, stems, roots, and nodules) were harvested 28 days after planting. Leaf material did not include petioles and stems did not include buds. Roots consisted of the entire root system with laterals. Several plants grown at the same time were pooled for each of the three biological replicates. Biological replicates were planted on separate days. Nodules were harvested from plants inoculated with *Sinorhizobium meliloti *strain 1021 one and seven days after sowing. Reproductive organs were harvested from plants that were vernalized for two weeks to decrease the time to flowering. Flowers were harvested on the day of opening. Pods were harvested from 1 to 21 days after the appearance of the floral bud to cover a wide range of developmental stages. Harvested plant material was frozen in liquid nitrogen before storage at -80°C.

### RNA isolation and cDNA synthesis

Total RNA was extracted using Trizol reagent [36], following the manufacturer's instructions (Invitrogen GmbH, Karlsruhe, Germany). RNA was quantified using a Nanodrop Spectrophotometer ND-100 (NanoDrop Technologies, Wilington, DE). Sixty μg of total RNA were digested with RNase free DNase1 (Ambion Inc., Houston, TX), according to manufacturer's protocol. RNA integrity was checked using an Agilent 2100 BioAnalyser and RNA 6000 Nano Chips (Agilient Technologies, Waldbronn, Germany), and by electrophoresis on a 3% (v/w) agarose gel before and after DNase I treatment. The absence of contaminating genomic DNA after DNase I treatment was verified by PCR analysis, using primer pairs designed to amplify a 107 bp genomic fragment of the control gene, *Ubiquitin *(TC102473intronF, 5'-GTCCTCTAAGGTTTAATGAACCGG-3'; TC102473intronR, 5'-GAAAGACACAGCCAAGTTGCAC-3').

First-strand complementary DNA was synthesized by priming with oligo-dT_12–18 _(Qiagen, Hilden, Germany), using SuperScript III reverse transcriptase (Invitrogen GmbH, Karlsruhe, Germany) following the instructions of the provider. To assess cDNA synthesis efficiency, qPCR was used to amplify segments in the 5' and 3' regions of *Ubiquitin *cDNA approx. 1600 and 400 bp from the 3'-end, respectively (primers: TC102473_5'F, 5'-TTGGAGACGGATTCCATTGCT-3'; TC102473_5'R, 5'-GCCAATTCCTTCCCTTCGAA-3; TC102473_3'F, 5'-GGCCCTAGAACATTTCCTGTGG-3'; and TC102473_3'R, 5'-TTGGCAACCAAAATGTTCCC-3'). If ΔCt (Ct3'-Ct5') < 2, then cDNA synthesis efficiency was judged to be satisfactory, and the cDNA was considered suitable for qRT-PCR analysis.

### PCR primer design

The primer design pipeline was implemented in object-oriented PERL modules supported by a MySQL relational database. Primers iterated through three phases before approval: design, specificity, and selection.

The design phase interrogated TF genes with a sliding window 250 bp across that stepped 50 bp along the entire target sequence, generating primer candidates at each window. Experimental conditions, as outlined in the Results section above, were enforced by the following MIT Primer3 parameters: PRIMER_MIN_TM 58, PRIMER_OPT_TM 60, PRIMER_MAX_TM 61, PRIMER_SELF_ANY 6, PRIMER_SELF_END 2, PRIMER_MAX_POLY_X 3, and PRIMER_PRODUCT_SIZE_RANGE '100–150' [37]. The specificity phase aligned primer candidates via WU-Blast to a database of all known Medicago sequences. The selection phase sorted primer candidates by the number of possible secondary hits, self-complementarity, and poly-X characteristics. Secondary hits were defined as specificity alignments that contained at least one of the terminal ends of the primer and achieved 80% or greater identity over the length of the primer. The sequences of each primer pair are given in Supplementary Material (see Additional file 1).

### Real-time PCR conditions and analysis

PCR reactions were carried out in an ABI PRISM^® ^7900 HT Sequence Detection System (Applied Biosystems, Foster City, CA, USA). SYBR^® ^Green was used to quantify dsDNA synthesis. Reactions (5 μl total volume) were performed in an optical 384-well plate containing 2.5 μl 2 × SYBR^® ^Green Power Master Mix reagent (Applied Biosystems, Warringen, UK), 5 ng cDNA and 200 nM of each gene-specific primer. Primer pairs were aliquoted using a pipetting robot (Evolution P3 liquid handling system, Perkin Elmer, MA, USA) to minimize pipetting errors. cDNA was aliquoted as a master mix of cDNA and 2 × SYBR^® ^Green reagent, using an electronic Eppendorf multipipette. Reaction plates were sealed with a transparent adhesive cover before proceeding (Applied Biosystems, Foster City, CA, USA). All templates were amplified using the following standard PCR protocol: 50°C for 2 min; 95°C for 10 min; 40 cycles of 95°C for 15 sec and 60°C for 1 min, and SYBR^® ^Green fluorescence was measured continuously. Melting curves were generated after 40 cycles by heating the sample up to 95°C for 15 sec followed by cooling down to 60°C for 15 s and heating the samples to 95°C for 15 sec.

Data analysis was performed with the SDS 2.2.1 software (Applied Biosystems). To determine the threshold cycle value (C_T_) for each PCR reaction, the threshold (Δ*R*_n_) was set within the logarithmic amplification phase. All amplification plots were analyzed with an Δ*R*_n _of 0.2. PCR efficiency (E) was estimated using LinReg software with data obtained from the exponential phase of each individual amplification plot and the equation (1+E) = 10^slope ^[31]. To compare data from different PCR runs and different cDNA samples, C_T _values were normalized against the geometric mean of four reference genes (*Ubquitin*, *PPRep*, *PDF2*, and *PTB*), whose transcript levels were most stable across the biological samples analyzed. The average of the geometric mean of these four genes for all 18 samples was C_T _21.23 ± SD1.15. For normalization, the mean reference gene C_T _value was substracted from the C_T _value of the TF gene of interest, yielding a ΔC_T _value. The expression ratios for the identification of organ-enhanced genes were obtained using the following formula on all 30 organ combinations: ${\left(1+\text{E}\right)}^{\Delta \Delta {\text{C}}_{\text{T}}}$, where ΔΔC_T _was calculated by ΔC_TA _minus ΔC_Tb_, A and B are averages of three biological replicates of the two organs being compared, and *E *is the PCR efficiency. Dissociation curves were analysed using SDS 2.2.1 software (Applied Biosystems). RT-PCR products were resolved on 3% (w/v) agarose gels (LE Agarose, Biozym, Oldendorf, Germany) run at 4 V cm^-1 ^in TAE Tris-Acetate-EDTA buffer, along with a 200-bp DNA-standard ladder (Promega GmbH). A subset of 178 RT-PCR products was sequenced at the JC Venter Institute (Rockville, MD, USA).

## Competing interests

The authors declare that they have no competing interests.

## Authors' contributions

KK performed the experimental work and helped draft the manuscript. W-RS, TC, and MS helped to conceive the project and provided practical advice. MW, FC, and CDT carried out bioinformatic analysis of Medicago TFs, and HW designed gene-specific primers. JCR and YX were responsible for the sequencing and analysis of the PCR products. IT carried out qRT-PCR and TG performed statistical analyses. MU designed and coordinated the project and wrote the manuscript.

## Supplementary Material

Additional file 1 Complete list of TF genes, primer sequences and corresponding PCR efficiencies. The TF primer platform was established based on the data for the gene models according to SA, specific amplification; NA, no amplification; NS, non-specific amplification. RT-PCR products of primer sequences indicated in bold were sequenced. Click here for file

Additional file 2 Complete list of TF genes, gene families and experimental data. Shown are the Medicago gene Accession Numbers (TIGR) in ascending order, the TC number if available, as well as the transcription factor family and the subfamily names. The next columns show experimental results for the real-time RT-PCR reactions performed on six different organs of Medicago in three indipendent biological replicates, as explained in the manuscript. CT = not normalized CT value, ΔCT = CT value normalized against the geometric mean of 4 house keeping genes; ΔΔCT = power(PCReff;-ΔCT); log2 ΔΔCT = logarithmus of ΔCT. Click here for file
